# Towards Chinese text and DNA shift encoding scheme based on biomass plasmid storage

**DOI:** 10.3389/fbinf.2023.1276934

**Published:** 2023-10-12

**Authors:** Xu Yang, Langwen Lai, Xiaoli Qiang, Ming Deng, Yuhao Xie, Xiaolong Shi, Zheng Kou

**Affiliations:** ^1^ Institute of Computing Science and Technology, Guangzhou University, Guangzhou, China; ^2^ School of Mathematical Science, Inner Mongolia University, Hohhot, China

**Keywords:** DNA storage, Chinese text storage, DNA shift coding, plasmid storage, DNA long double-stranded structure storage

## Abstract

DNA, as the storage medium in organisms, can address the shortcomings of existing electromagnetic storage media, such as low information density, high maintenance power consumption, and short storage time. Current research on DNA storage mainly focuses on designing corresponding encoders to convert binary data into DNA base data that meets biological constraints. We have created a new Chinese character code table that enables exceptionally high information storage density for storing Chinese characters (compared to traditional UTF-8 encoding). To meet biological constraints, we have devised a DNA shift coding scheme with low algorithmic complexity, which can encode any strand of DNA even has excessively long homopolymer. The designed DNA sequence will be stored in a double-stranded plasmid of 744bp, ensuring high reliability during storage. Additionally, the plasmid‘s resistance to environmental interference ensuring long-term stable information storage. Moreover, it can be replicated at a lower cost.

## 1 Introduction

With the exponential growth of internet data, the demand for efficient and high-quality storage media is increasingly evident (Zhirnov et al., 2016)^.^ Mainstream storage media include magnetic storage, optical storage, and semiconductor storage (Kun et al., 2020), all of which suffer from issues such as short storage lifespan and the need for high energy consumption to maintain stored content (Williams et al., 2002; Goda and Kitsuregawa, 2012; Extance, 2016; Panda et al., 2018). For instance, astronomical data records require the use of tons of heavy hard drives, and the lifespan of these hard drives is limited by their read/write cycles. Moreover, copying and transferring stored data consume significant amounts of time and electricity (Ritter and Society enneotAC, 2015; Organick et al., 2018; Rutten et al., 2018). According to research by Huawei’s Sweden Research Institute, by 2030, the energy consumption of global data centers is expected to account for 3%–10% of the total electricity usage.

China currently holds the position as the world’s largest internet user base, and the Chinese characters boast the highest number of speakers globally. It is also the oldest continuously used writing system, with an extensive historical legacy. Chinese characters have been the primary official script throughout the dynastic periods in China. In ancient times, Chinese characters served as the sole medium for international communication in the East Asian region. Even up until the 20th century, Chinese characters still served as official written norms for countries like Japan, the Korean Peninsula, Vietnam, and Ryukyu. Chinese characters are also currently preserved as the most abundant form of recorded writing in human history. However, due to limitations of storage media, the vast majority of historical events in human civilization were recorded and transmitted using text, resulting in low efficiency in storage and retrieval, as well as poor resistance to environmental interference. Consequently, only a small fraction of events experienced by a limited number of people have been recorded in history. Nevertheless, with the advancement of technology, new storage media like high-definition videos possess significantly greater information-carrying capacity than traditional written records. They can convey a larger volume of information within a given period of time.

Living systems possess remarkably high storage capacity and low maintenance costs for genetic information, making DNA one of the popular choices for the “next-generation information storage medium” (Hakami et al., 2015; Yazdi et al., 2015). Predictions suggest that the data storage density of DNA (Deoxyribonucleic Acid) molecules is at least seven orders of magnitude higher than current mainstream storage methods like magnetic storage, optical storage, and semiconductor solid-state storage. When combined with its inherent high stability (the storage medium does not degrade with read cycles) and exponential replication mechanism (efficient and low-energy data copying), DNA storage holds promise in addressing issues related to massive data storage, maintenance, and transfer (Dietrich and Been, 2001; Garzon et al., 2004; Cui et al., 2007; Bornholt et al., 2016; Xu et al., 2020). DNA storage technology converts digital information into a DNA sequence composed of adenine (A), guanine (G), thymine (T), and cytosine (C) and then retrieve the data by reading the DNA sequence to restore the original digital information (Bonnet et al., 2010). Compared to traditional storage media, DNA oligonucleotides, as a storage medium, offer higher storage density and longer storage duration (Allentoft et al., 2012; Grass et al., 2015). Research indicates that 1 mL of DNA oligonucleotide solution can store over 10,000 gigabits of data, and under certain conditions, the storage time of DNA can extend to hundreds of years or even longer (Kim et al., 2004; Miller et al., 2012; Orlando et al., 2013; Anavy et al., 2019).

In 1997, Joe Davis proposed an intriguing experiment aiming to store digital information in DNA molecules (Davis, 1996) and embed them into the chromosomes of bacteria (Cremer and Cremer, 2010). The experimenters utilized a “gene gun” device, which could shoot DNA sequences into cells. Once the DNA sequence was injected into the cell, its DNA repair mechanism would integrate the new DNA sequence into its chromosomes. However, due to the technological limitations of that time, the experiment was unsuccessful. Nevertheless, this experiment sparked the creativity of many scientists and artists, driving the development of DNA storage technology. In 2003, Wong et al. introduced a method using artificial DNA sequences to store and retrieve information (Wong et al., 2003). The authors proposed a technique to encode meaningful information into DNA sequences and insert them into living hosts such as bacteria, insects, or plants, enabling very long-term data preservation. In 2013, Goldman et al. combined Huffman coding, fourfold overlap, and ternary encoding methods with DNA (Goldman et al., 2013). In their experiment, the Goldman team successfully stored 739 kB of content in DNA, occupying significantly less space than traditional digital storage media. They also demonstrated that this DNA storage technology could be stored at room temperature for long periods and accurately decoded and read. This research indicates that using DNA as an information storage medium can significantly increase storage density, reduce storage costs, and achieve long-term information preservation and efficient information retrieval. In 2017, Erlich et al. introduced the “DNA Fountain” into the field of DNA storage, a method with high information density, long-term stability, low storage costs, and efficiency advantages (Erlich and Zielinski, 2017). The “Fountain” refers to using a certain encoder to generate an infinite number of droplet data while employing the Luby transform and screening the generated droplet data using a certain encoder (Luby, 2002; MacKay, 2005). In 2018, researchers from Microsoft Research and the University of Washington proposed a random access technique for DNA storage systems, allowing for rapid and accurate locating and retrieving of stored data in large-scale DNA storage systems (Organick et al., 2018). That same year, Anavy et al. used composite DNA letters in the DNA storage domain, achieving higher information capacity (Anavy et al., 2018). In 2019, Choi et al. introduced an enhanced encoding character method for DNA data storage, using degenerate bases to expand the DNA encoding character set and increase information capacity (Thoo and Brown, 1992; Choi et al., 2019). In the same year, a team from Tianjin University encoded “The Analects of Confucius” into DNA plasmids. However, due to the direct compilation of machine code, the encoding density was relatively low, at only 0.85 bit/nt. In 2020, Zhang et al. introduced a DNA storage strategy based on Base64 encoding (Zhang et al., 2020). In this strategy, certain text information was encoded into DNA sequences through Base64 encoding, code reshaping, balancing, and data mapping. The synthesized DNA molecules were then inserted into circular plasmids for long-term information storage (Josefsson, 2006). The introduction of balanced codes effectively controlled the GC content and prevented consecutive base repetitions, which is crucial for minimizing errors during DNA synthesis and sequencing. In 2022, Zhi et al. drew inspiration from Chinese traditional culture, Yin and Yang, and proposed a powerful DNA encoding method called the “Yin-Yang Encoder” (Ping et al., 2022). This encoding method used two rules to encode two binary bits into a nucleotide, resulting in a DNA sequence with balanced GC content and no consecutive homopolymers. The authors encoded two representative file formats and stored them externally. Experimental results showed that the Yin-Yang Encoder exhibited high robustness and reliability for various data types.

Based on the above, current research on DNA storage mainly focuses on designing encoders, which can convert data into DNA sequences that satisfy biological constraints for reliable and high-density storage. The design goals of encoders mainly aim to achieve higher coding efficiency, stronger error-correction capabilities, and higher information coding density. While the current encoding methods can effectively convert information, there is still considerable potential for improving coding efficiency and information density. For example, the use of fountain codes with LT encoding and LFSR, as well as the utilization of PRNG, can greatly impact the coding efficiency, leading to a limited generation of usable information in the seed coding space. Moreover, the encoders must guarantee the primers’ uniqueness, otherwise wrong amplifications will occur in the PCR process, thus reduce the data payload information density. Erlich et al. stored information like the “DNA hard drive”, if a primers’ encoding space is use up, new primer sequence is need. Peter Michael Schwarz et al. used the T7 promoter to physically separate primers from the stored information, increasing information capacity. DNA as the storage medium in biological storage, various biochemical constraints must be considered during synthesis, storage, sequencing, PCR, and other processes (Chen and Seeburg, 1985; Benjamini and Speed, 2012) to reduce the probability of errors. Among them, two important biochemical constraints are GC content and homopolymer length. GC content refers to the ratio of the number of G and C base pairs to the total number of bases in a DNA sequence. GC content should be maintained between 40% and 60%. DNA with excessively high or low GC content is susceptible to thermodynamic effects, leading to increased instability. Homopolymer length refers to the consecutive bases in a DNA molecule. Longer homopolymers (e.g., consecutive bases exceeding 4) are susceptible to the influence of physical and chemical factors, leading to increased instability in DNA and the formation of hairpin structures. Therefore, in biological research, the length of consecutive homopolymers is a significant biochemical constraint used for studying DNA quality and analyzing the stability of DNA fragments, among other considerations. To ensure the quality and stability of DNA sequences, biologists must adhere to strict biochemical constraints, and the length of consecutive homopolymers should not exceed four nucleotides (nt).

Drawing inspiration from base mutation, we have devised a DNA shift encoder capable of incrementally designing base bit mutations while adhering to biological constraints. The DNA shift encoder boasts remarkably high encoding efficiency, enabling the encoding of greater information content in a shorter timeframe than other encoders.

Recently, scholars have reported using storage methods based on degenerate bases. This approach allows for higher information capacity compared to traditional encoding methods. However, it may lead to additional costs in sequencing. In this paper, our DNA storage approach remains aligned with the conventional use of the four base letters: A, T, C, and G, without incorporating degenerate bases.

For the DNA storage of Chinese text, the commonly used computer code table is UTF-8 (8-bit Unicode Transformation Format), with a scale of 24 bits, capable of accommodating 16,777,216 characters (Yergeau, 2003). However, the scale of commonly used Chinese characters is approximately 5,000 characters, which is much smaller than the capacity of UTF-8, indicating significant redundancy when storing common Chinese characters using UTF-8 (Chu et al., 2012). This paper proposes a customized encoding scheme for Chinese text, establishing a universal GB2312 code table (including 6,763 Chinese characters and 682 non-Chinese graphic characters) and a mapping between the code table and DNA bases, converting Chinese characters into DNA base sequences. Additionally, an innovative DNA shift encoding method is proposed, which can efficiently accomplish data hybridization for information encoding, ensuring smooth passage through biological constraints. As for the storage method, plasmids are utilized as the secondary carrier for storing information. Plasmid is double-stranded circular structures, offering strong stability during the storage process and high efficiency in information replication and sequencing. They can also be embedded in living organisms like *Escherichia coli* for biological conditions cultivation, enabling efficient and low-cost information replication and storage. Furthermore, plasmids can be enveloped in protein shells to enhance the storage stability of the information and increase their resistance to environmental interference. Plasmids enveloped in protein shells will exhibit higher affinity with living microorganisms, resulting in stable, long-lasting, and low-power information storage.

## 2 Materials and methods

**FIGURE 1:**
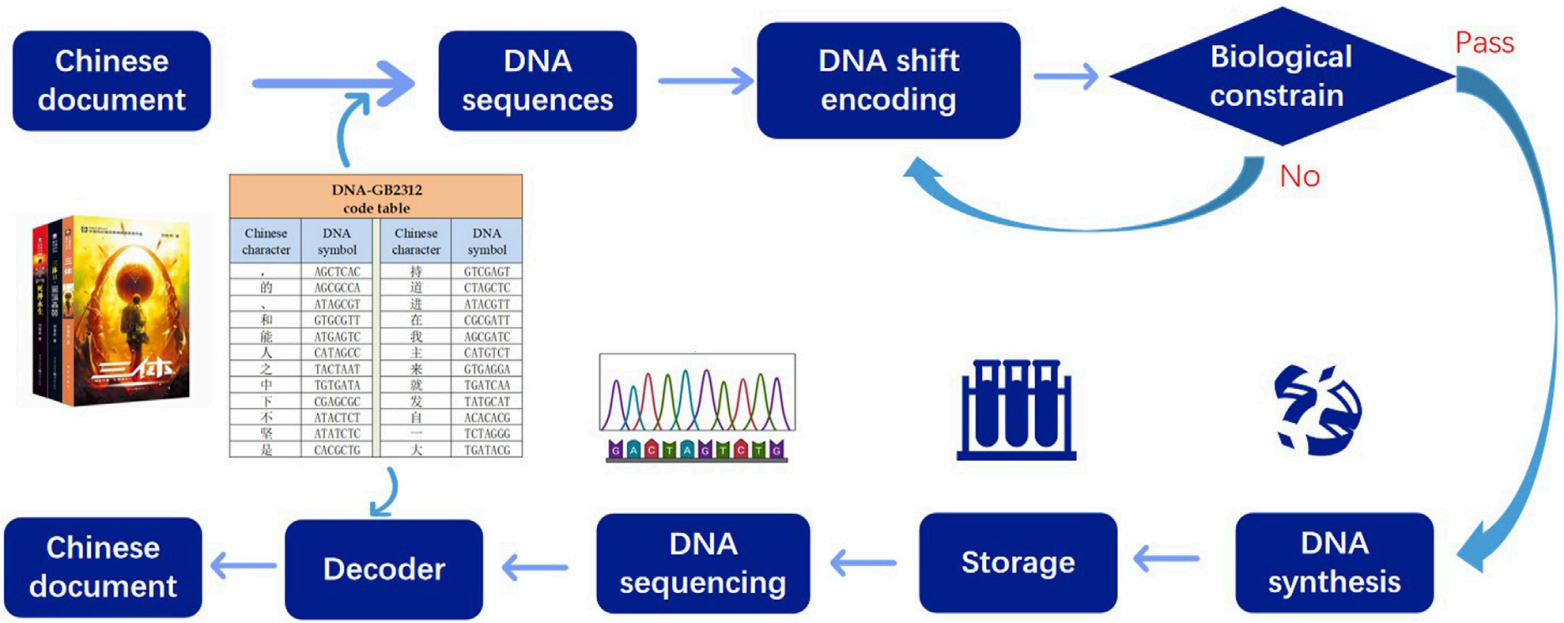
DNA storage flow chart. Firstly the Chinese characters are converted into DNA sequences according to the DNA-GB2312 code table. After chain shift transformation, the DNA sequences meet the biological constraints, and then the sequences are stored. When decoding, the sequences are sequenced and decoder is injected.

Given the extensive utilization of Chinese text on the contemporary Internet, alongside its historical significance as one of the most widely employed and meticulously preserved writing systems, our attention turns toward the DNA storage of Chinese text. The primary process of storage involves the following steps: In the first step, convert the Chinese text to DNA sequences based on the DNA-GB2312 code table. In the second step, the DNA sequences undergo a DNA shift algorithm to ensure that the DNA chains satisfy biological constraints. The third step involves adding the parameters used in the transformation algorithm and the RS error correction code to the transformed DNA sequences. Finally, the transformed DNA sequences, along with the additional information, are synthesized and stored using plasmids. The flow chart depicted in Figure 1 illustrates this process.

The decoding process involves the following steps: The stored DNA is subjected to sequencing to retrieve the encoded information. Using the RS error correction code, the system checks if any errors occurred during the storage or retrieval process. If errors are detected, the system performs error correction to ensure data accuracy. Next, the DNA sequences undergo a DNA shift algorithm using the provided parameters to reverse the transformations applied during the encoding process, recovering the original DNA chain information. With the recovered DNA chain information, the system employs the DNA-GB2312 code table to translate and decode the information back into the original Chinese text.

### 2.1 DNA-GB2312 code table design

Currently, the widely adopted character encoding for computers is UTF-8, which uses 24 bits and includes 16,777,216 characters. However, the commonly used Chinese characters are approximately 5,000 characters in total. Relying solely on the UTF-8 character set would lead to low information density when encoding Chinese characters. To address this issue, we have chosen to use the Information Exchange Chinese Character Encoding Character Set-GB2312 character set (which includes 6,763 Chinese characters and 682 non-Chinese graphic characters). We establish the DNA-GB2312 code table by using a 7-bit base to represent one Chinese character.

The size of the DNA code table is related to the length of the DNA short sequences. We denote the length of a sequence in the DNA code table as “n” and the maximum capacity of the DNA code table as “M”. Their relationship can be expressed by Eq. 1.

$$M=4^{n}$$ (1)

The creation of a DNA code table involves several important steps. Firstly, it is essential to determine the number of characters, denoted as “K” that will be included in the code table. It is crucial to ensure that K is smaller than the maximum capacity, “M” of the DNA code table to allow for the inclusion of all characters. This ensures that the code table can effectively accommodate all the characters intended for storage. Once the number of characters, K, is determined, the next step is calculating the DNA sequence length, denoted as “n” required for the code table. This can be achieved using Eq. 2

$$M=4^{n}\geq K$$ (2)

In this context, the value of “n” is typically chosen to be the smallest possible, which helps reduce unnecessary waste. For instance, when creating a DNA code table for 16 characters, “n” could take on values such as 2, 3, 4, 5, 6, and so on, representing larger positive integers. However, the optimal approach is to set “n” as 2, which requires only two nucleotides to precisely construct the code table for all 16 characters.

We have pre-created the DNA-GB2312 code table with K = 7445 and *n* = 7. Calculations indicate that M = 16384, resulting in a redundancy of 8,939 bits in character space. This redundancy is intended for customized document storage. The customization process begins by obtaining the Chinese text to be stored. The algorithm will automatically recognize the characters present in the document. If a character is found in the DNA-GB2312 code table, it will be filled using the corresponding sequence from the code table. If a character is absent in the code table, the algorithm will allocate a suitable nucleotide sequence from the available redundant space. This process maps the character to a corresponding DNA sequence. The mapping relationships will be stored in an array, which will function as the decryption key during the decoding process. Finally, the input characters are traversed, and the characters are combined with the corresponding DNA short sequences from the array to form the code table.

### 2.2 DNA shift encoding scheme

**FIGURE 2:**
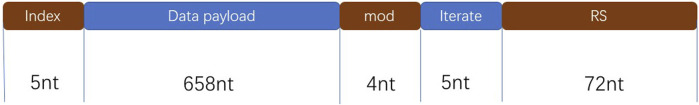
Sequence structure.

After converting the Chinese document into the corresponding DNA nucleotide sequence, the Encoder will split the DNA sequence into short DNA sequences according to certain requirements. Then, an address index “index” is set at the beginning of the sequence to form the DNA sequence to be transformed. Subsequently, the “iterate”, “mod”, and RS error correction codes are added to the DNA sequence, forming the sequence structure depicted in Figure 2. The DNA sequences under scrutiny might consist of 4 consecutive nucleotides or exhibit irregular GC content, thereby contravening biochemical constraints. In the fountain code, chains that do not meet the biological constraints will be directly discarded, resulting in a lower information density of the encoding. We have added “mod” and “iterate” bits to enhance the information density. Through DNA shift encoding, as many chains as possible are transformed into ones that satisfy the biological constraints successfully, thereby increasing the coding information capacity. Additionally, the core of the DNA shift encoding lies in cyclic modulo verification, which contributes to its lower algorithm complexity. The time complexity of the encoding process is O(n), making it efficient and practical.

DNA shift encoding is inspired by the Caesar cipher (Goyal and Kinger, 2013), an ancient encryption technique that originated during the time of the Roman Empire and was used by the Roman general Caesar, hence its name. The Caesar cipher is a simple substitution cipher that shifts each letter in the plaintext by a fixed offset to obtain the corresponding ciphertext. The encryption algorithm for the shift cipher can be represented by a straightforward mathematical formula, as shown in Eq. 3.

$$V\_i=\left(P\_i+k\right)\%26$$ (3)

In the given context, “ V_i ” represents the ciphertext of the i character, “ P_i ” represents the plaintext of the i character, “k” denotes the shift offset, and 26 represents the number of letters in the English alphabet, which is used to map the encrypted characters back to the alphabet. For example, if the offset is 2 and the plaintext is “ABCDE”, the ciphertext obtained after the shift is “CDEFG”.

The decryption process is equally straightforward. To decrypt the ciphertext, you need to shift each letter in the ciphertext in the opposite direction of the encryption shift offset. This means shifting the letters backward in the alphabet. For instance, in the Caesar cipher with a shift offset of 2, during the decryption process, each letter in the ciphertext needs to be shifted two positions to the left, effectively moving the entire alphabet two positions to the left to obtain the plaintext.

DNA shift encoding involves transforming certain bases in a DNA sequence into another type of base according to specific rules. For example, it can convert a DNA sequence containing six consecutive homopolymers, “AAAAAA”, into a sequence that does not have consecutive homopolymers, such as “AAAGAA” to ensure the DNA sequence meets biochemical conditions. The shift encoding used in this paper requires using two equations: Formulas 4, 5.

i=iterate % mod (4)

Z=i×j (5)

In the given description, Formula 4 represents an equation with “iterate” as a positive integer starting from 2 and incrementing upwards and “mod” as a positive integer starting from 3 and increasing sequentially. Formula 5 introduces the variable “j” which is a positive integer starting from 0 and incrementing upwards. The “iterate” value is taken modulo “mod” resulting in the remainder “i” and “Z” being defined as the multiples of “i”. The process involves incrementing “iterate” from 2 in the positive integer direction and taking its modulo with “mod” after each increment. Upon obtaining the remainder “i” the value of “Z” is recalculated. After evaluating Formula 5 to obtain a value for “i” the variable “j” resets to 0 and increments in the positive integer direction until its value exceeds the length of the target DNA sequence. Once the increment of “j” is completed, a series of multiples of “i” values for “Z” is obtained. The series of positive integers represented by “Z” obtained from the increment of “j” indicates the positions in the target DNA sequence that require transformation. For example, if “Z” is 0, it means the first base in the target sequence needs to be transformed; if “Z” is 20, the 20th base in the target sequence needs to be transformed. This transformation process is designed to convert existing continuous bases into non-continuous bases.

**FIGURE 3:**
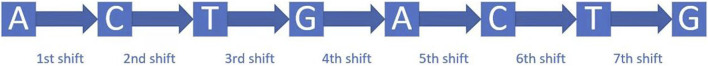
Conversion example.

The “iterate” value, when taken modulo “mod” produces a value denoted as “i” By calculating multiples of “i” a series of “Z” values is obtained. It is important to note that “iterate” cannot be a multiple of “mod” or a multiple of “mod” plus one. The process of continuous DNA shift encoding is applied to the generated DNA sequence. Subsequently, the DNA sequence is subjected to biological constraints such as polymer length (<4) and GC content (40%–60%). If the target sequence fails to meet the biochemical conditions, the “iterate” value increases by one. This process continues until the “iterate” value exceeds 4 times “mod” plus 2. At this point, the “iterate” value stops incrementing, the “mod” value increases by one, and “iterate” starts incrementing again from 2. DNA contains four bases: A, C, T, and G, which can be considered a quaternary system. After four transformations, A will return to A, as shown in Figure 3. The transformation positions in the DNA sequence, denoted by “Z” are determined by “i” which is obtained by taking “iterate” modulo “mod” This means that for a given “mod” value, to obtain the same “i” value, there can be many different “iterate” values. Therefore, the transformation cycle is determined by “mod” and its transformation cycle is 4 times “mod”.

Because “iterate” starts from 2 rather than incrementing from 0, a complete transformation cycle is achieved when the value of “iterate” reaches 4 times “mod” + 2. If “iterate” continues to increment, the DNA sequence obtained after transformation will repeat a previously encountered DNA sequence. Thus, the resulting DNA sequence still does not meet the biochemical conditions. Therefore, the strategy at this point is to increase the value of “mod” by 1 and reset “iterate” to 2, effectively refreshing the transformation parameters.

### 2.3 Reed-solomon code

The Reed-Solomon Code (RS code) is a mathematical formula defined on a finite field F and a polynomial ring F(x). Let n and k satisfy $1\leq k\leq n\leq \left|F\right|$ , where |F| represents the number of elements in the finite field F. We denote n-determined elements in F as $\left\{x_{1},x_{2},\ldots ,x_{n}\right\}$ . The codeword C is obtained by evaluating polynomials in F for each x_i_ such that the polynomial degree is less than k, as shown in formula 6.

$$C=\left\{\left(f\left(x_{1}\right),f\left(x_{2}\right),\ldots ,f\left(x_{n}\right)\right)|f\in F\left[x\right],\deg\left(f\right)<k\right\}$$ (6)

The codeword C is an $\left[n,k,n-k+1\right]$ code, meaning it is a linear code (Norouzi et al., 2012) over the finite field F with length n, dimension k, and minimum Hamming distance of n-k+1. In practice, Galois fields are commonly used as finite fields to implement operations of the RS code (Benvenuto, 2012). The number of parity check codes in the RS code depends on the data length and error-correcting capability, usually requiring more check codes than the data length. RS code can detect and correct errors when a few errors occur during transmission. However, if the number of errors exceeds its error-correcting capability, the RS code may fail to recover the original data. In DNA storage, DNA sequences can be affected by chemical, physical, and biological factors, leading to mutations and inaccuracies. Many scientists have introduced RS code into DNA storage research to address these potential errors.

### 2.4 DNA shift decoding scheme

Decoding a DNA sequence requires three steps. First, the DNA sequence is corrected to ensure that the DNA sequence is correct. Second, reverse transforming the DNA sequence so that the DNA sequence is restored back to the DNA sequence that was not transformed at the time of encoding, and checking the DNA code table to obtain the characters stored corresponding to that strand. Finally, each strand is sorted, and the characters on the DNA splicing strand are restored to the original file.

Specifically, the decoding process involves three main steps. In the first step, the DNA sequence to be decoded is converted into binary form. Then, the RS error correction is applied to the DNA sequence to detect and correct any errors in DNA fragments. This ensures that subsequent decoding will not encounter garbled or failed results. In the second step, the decoding process considers the DNA’s mod value, iterate value, and RS code. It performs a chain-shifting decoding transformation based on equations 4 and 5. The iteration starts with j = 0, incrementing j in the positive integer direction until it exceeds the length of the DNA sequence to be transformed. At that point, the iterate value is reduced by 1. After the decrease in the iterate value, j starts again from 0 and continues incrementing. This process continues until the iterate value decreases to 2, at this point the mod value is reduced by 1, and the iterate value becomes 4 × mod + 2. The reverse transformation is completed when the mod value is 3 and the iterate value is 2. The decoding process involves rules opposite to those used during encoding, where A, G, T, and C are transformed into G, T, C, and A, respectively. This reversal ensures the correct restoration of the DNA sequence as it was originally encoded. After the DNA sequence is transformed back to its original encoded form, it must be separated. The DNA sequence is divided into index and data blocks and then concatenated to complete the decoding process.

In the third step, DNA sequences have been transformed into decimal numbers, each corresponding to a text segment. These decimal numbers are sorted. Subsequently, the text corresponding to the decimal numbers is concatenated in ascending order. At this point, the entire decoding process is completed.

### 2.5 Biochemical experiment

Through biological experiments, he practical feasibility of this storage scheme is validated. The biological experiments for DNA storage primarily involve three steps: Firstly, the DNA sequences encoding the Chinese text are designed, and the encoded DNA sequences are synthesized to form DNA chains. Secondly, after the synthesis is completed, Sanger sequencing is employed to verify the consistency between the synthesized DNA and the target sequence. Additionally, the DNA chains are incorporated into plasmids. To ensure the accuracy of the synthesis, agarose gel electrophoresis is conducted to confirm the integrity of the target plasmids. The plasmid’s double-stranded circular structure is cut using restriction endonucleases, and the synthesized DNA chains are inserted into the plasmids, followed by ligation and annealing to form circular DNA molecules. Next, the DNA chains are stored. When the target information needs to be extracted, Sanger sequencing is used to obtain sequencing results for the target sequences between the plasmid sequencing primers. Finally, the sequencing results are decoded, successfully retrieving the stored Chinese text content.

Currently, most DNA synthesis is accomplished using PCR synthesizers. PCR synthesizers utilize Polymerase Chain Reaction (PCR) technology, which allows for the assembly of short DNA strands into longer chains, resulting in the generation of a substantial amount of DNA sequences ranging from 500 bp to 800 bp (Carr and Church, 2009; Shendure and Aiden, 2012). The process of DNA sequence synthesis mainly involves four steps: PCR amplification, enzymatic digestion, ligation, and transformation (Schochetman et al., 1988). Utilizing plasmids as biological storage vectors significantly enhances storage stability and resistance to environmental disturbances. Plasmids possess a double-stranded circular structure, which effectively mitigates the risk of base mutations during the storage process caused by electromagnetic radiation or changes in solution properties. Moreover, plasmids can efficiently replicate within living organisms. When encapsulated with a protein shell, plasmids further improve their storage longevity and affinity with living organisms.

## 3 Results

### 3.1 Simulation results

A DNA code list was created in the computer, through which multiple copies of Chinese texts were stored in the simulation, and all of these Chinese texts could be successfully encoded and decoded as shown in Table 1. In the encoding stage, all Chinese text contents could be encoded to generate DNA sequences. The Chinese characters in the texts were all found on the DNA code table, and there was no error in failing to find the character on the DNA code table. There is also the fact that all the DNA sequences generated by the encoding were of the same length, and after further testing and certification, it was observed that their GC content was between 40% and 60%, containing at most three consecutive homopolymers. At the decoding stage, all DNA sequences are capable of being decoded into Chinese text.

**TABLE 1:** The Simulation Result of DNA shift Encoding Scheme.

Chinese text	Text size (MB)	Number of words	Homopolymer (nt)	GC content (%)	Density (bits/nt)
家	0.49	292000	3	51	3.45
三体	2.59	800000	3	48	3.43
平凡的世界	1.56	837856	3	49	3.44

### 3.2 GC content and homopolymer analysis

**FIGURE 4:**
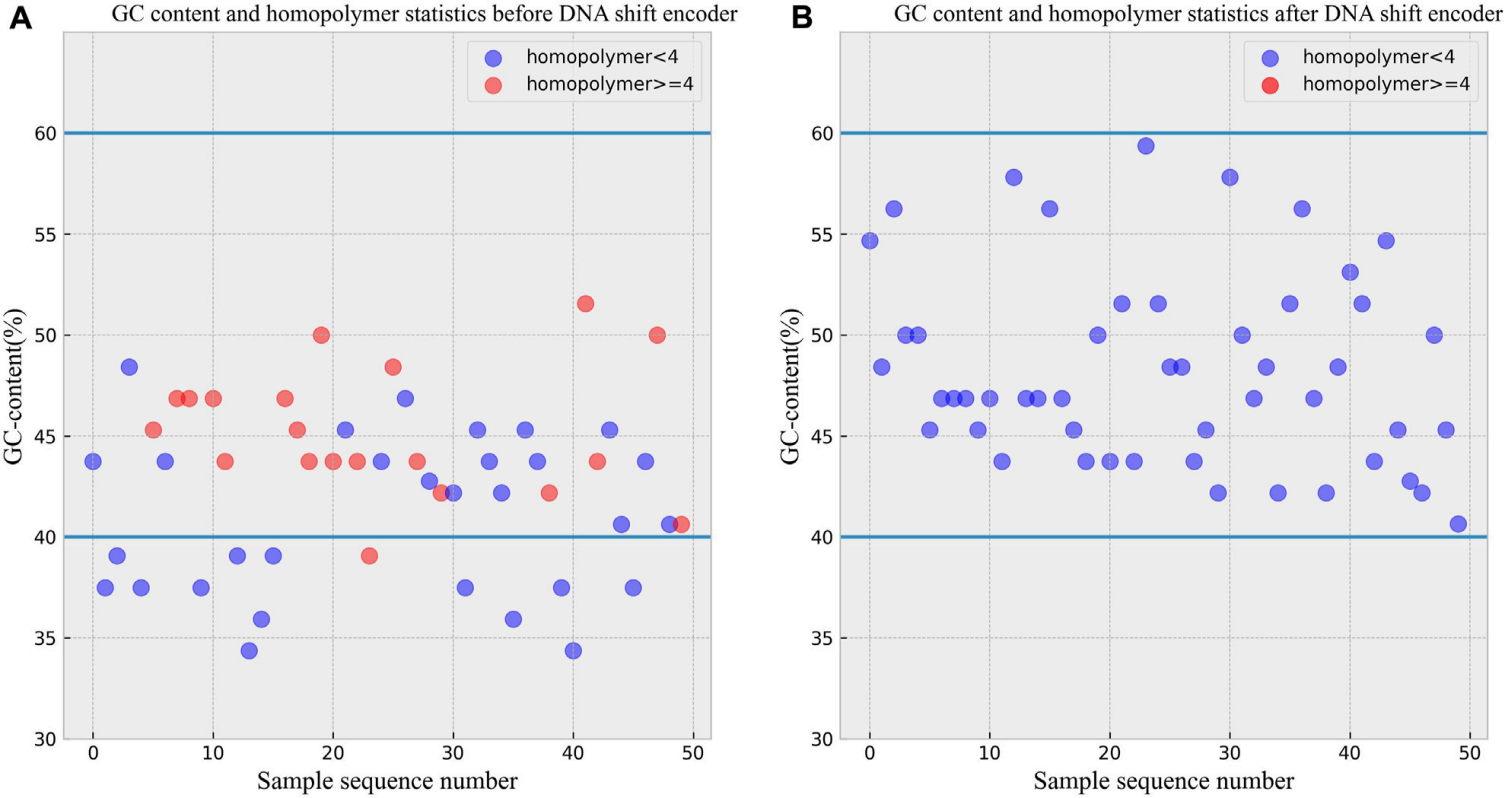
**(A)** GC content and homopolymer statistics before DNA shift encoder. **(B)** GC content and homopolymer statistics after DNA shift encoder.

To validate the ability of the DNA shift encoding technique to maintain GC content within the range of 40%–60% and can avoid consecutive tetranucleotide runs, statistical analyses were conducted on data before and after the transformation. A set of 50 DNA sequences was obtained by encoding a Chinese text, and the pre-transformation data is shown in Figure 4A. The data after DNA shift encoding is presented in Figure 4B. As evident from the figures, all 50 DNA sequences after the transformation maintained GC content within the desired 40%–60% range, and none of the sequences contained consecutive tetranucleotide runs. The encoder effectively transformed the sequences that did not adhere to biochemical constraints before encoding into DNA sequences that now comply with these constraints.

According to the experimental data before and after the transformation, it can be seen that the DNA storage scheme proposed in this paper can solve the problems of uneven GC content and the problem of containing continuous homopolymers, which proves that the DNA sequences generated after the DNA storage scheme proposed in this paper can be used in biological experiments, and there exists experimental feasibility.

### 3.3 Information capacity analysis

In order to assess the performance of the DNA storage method proposed in this paper compared to previous DNA storage methods, an information capacity analysis was conducted. To ensure the rigor of the experiments, DNA sequences of similar lengths to other classical DNA storage methods were designed. The DNA code table used in this study was based on the Chinese international standard GB2313, encompassing all characters from GB2313 and additional commonly used characters. Ultimately, the DNA code table consisted of 7824 characters, leading to a DNA sequence length of 7 nt in the designed code table. The DNA code table has been made available on GitHub (https://github.com/github-plus/DNA-/blob/master/char_dna.xls), where the first column represents Chinese characters, and the second column contains the corresponding DNA sequences. The index length was set to 8 nt, the data length was 105 nt (containing 15 characters), the mod was 3 nt, iterate was 4 nt, and the RS code was 8 nt, resulting in a final DNA sequence length of 128 nt. A computer simulation was used to store a 20463Bytes-sized Chinese text containing 6,821 characters, generating 455 DNA sequences. The theoretical information capacity of this approach is 3.42 bits/nt.

For the superiority of the DNA storage scheme proposed in this paper in storing Chinese, it is compared with other earlier DNA storage schemes (Church et al., 2012), as shown in Table 2.

**TABLE 2:** Comparison of DNA storage solutions.

	homopolymer (nt)	GC content (%)	Density (bits/nt)
Church et al. (2012)	3	2.5–100	1
Goldman et al. (2013)	1	22.5–82.5	1.58
Erlich and Zielinski (2017)	4	40–60	1.98
Choi et al. (2019)	3	0–100	3.37
Ping et al. (2022)	4	40–60	1.95
Base 64 (Zhang et al., 2020)	4	45–55	1.77
This work (DNA shift)	3	40–60	3.42

Note: DNA information capacity calculations are compared to general-purpose computer Chinese character storage space.

Comprehensive comparisons of the present storage scheme with previous classical papers and comparisons of using DNA code tables with and without DNA code tables show that the method proposed in this paper is superior to previous DNA storage methods when storing Chinese text.

### 3.4 Time complexity analysis

We analyze the time complexity of strand DNA shift encoding with the current mainstream DNA storage encoding: the DNA Fountain and Yin-Yang Code (Ping et al., 2022).

**FIGURE 5:**
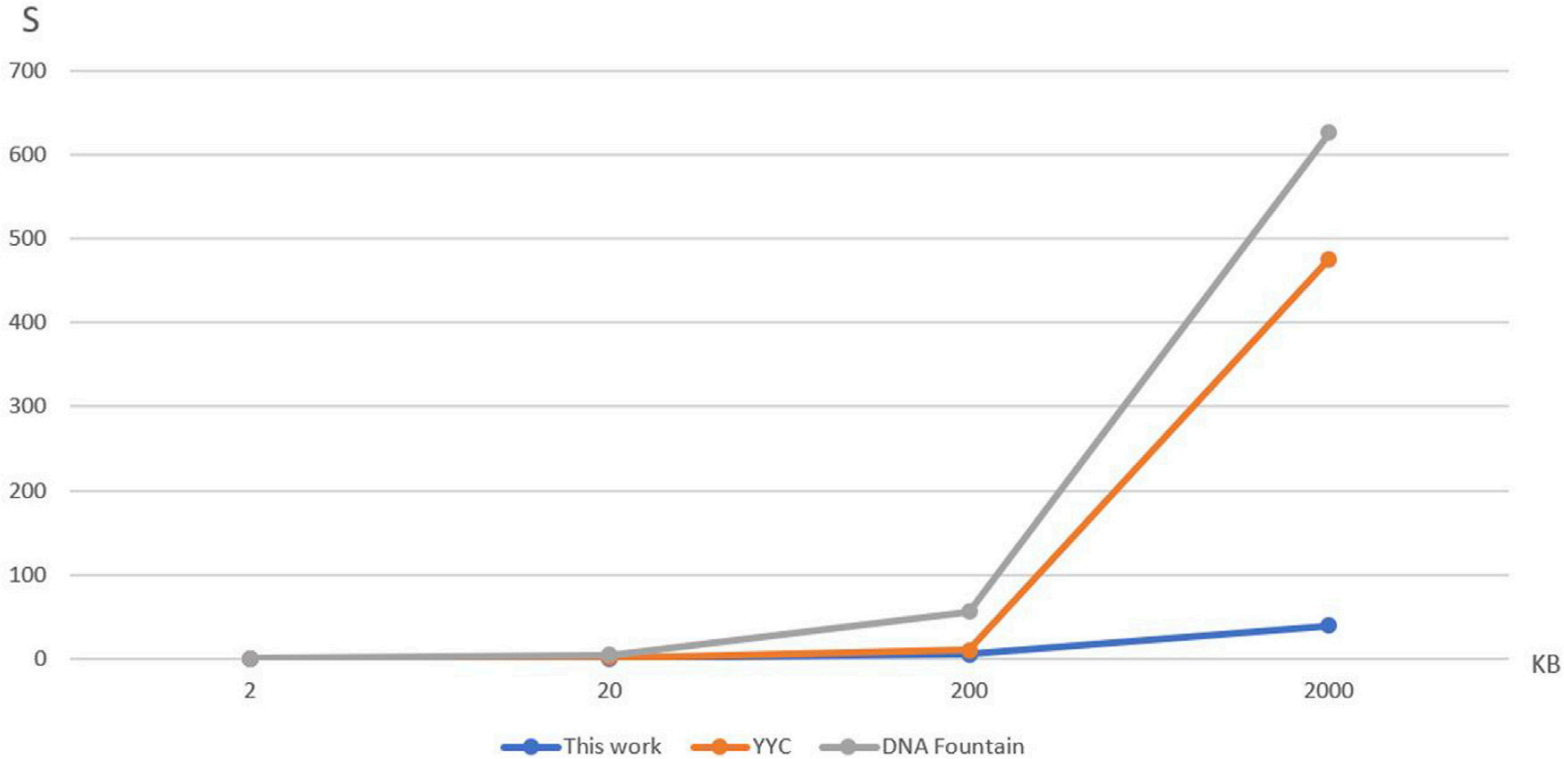
Comparison of time spent on DNA storage coding.

In order to verify the comparison of their encoding time at different data volumes, the samples are designed as four data volumes of Chinese text files, respectively, 2Kbyte, 2Mbyte, 20Mbyte, and 2Gbyte. In order to ensure the rigor of the experiment to prevent the impact of the encoding time because of the problems of a particular file and the design of each size, respectively, there are four different kinds of files. The time complexity analysis is run under the same computer equipment, CPU is R7-5800H and running memory is dual-channel 16G, Python version 3.7. The results obtained are shown in Tables 3, 4; Figure 5 below.

**TABLE 3:** Comparison of time (sec) of three storage schemes at different data volumes.

Text size	2 KB	2 MB	20 MB	2 GB
DNA Fountain (Erlich and Zielinski, 2017)	0.51	1648	-	-
YYC (Ping et al., 2022)	0.14	462	21389	-
This work (DNA shift)	0.14	39	8478	175,592,953

**TABLE 4:** Comparison of coding time increase.

	2∼20 Kbytes	20∼200 Kbytes	200∼2000 Kbytes
DNA Fountain (Erlich and Zielinski, 2017)	11.97	12	11.12
YYC (Ping et al., 2022)	5.19	9.17	47.6
This work (DNA shift)	4.64	8.6	7.8

To compare the encoding time of the three storage methods at different data volumes, Table 3 and Figure 5 were created. The encoding time represents the average time taken by each storage method to encode four files of varying data sizes. It can be observed from the results that the encoding time of YYC is lower than DNA Fountain for all four data volumes. Moreover, the proposed DNA storage method exhibits an even lower encoding time than YYC. Additionally, when storing a 2Gbytes text, both DNA Fountain and YYC encoding methods fail to find a solution within a feasible time frame.

In order to speculate which storage method performs better when storing larger files, a comparison of encoding time increments was conducted for the three storage methods. The encoding time for a large data volume is denoted as T_1_, and the encoding time for a small data volume is denoted as T_2_. The increment factor K is calculated according to Eq. 7.

$$K=\frac{T_{1}-T_{2}}{T_{2}}-1=\frac{T_{1}}{T_{2}}$$ (7)

The algorithm complexity of the proposed DNA storage method is O(n). Both DNA Fountain and YYC are also processes that generate data in a loop. However, DNA Fountain and YYC often encounter encoding inefficiencies due to their strategy of discarding non-compliant chains that do not meet biological constraints and continuously generating new chains. In contrast, the DNA shift encoding employed in the proposed method transforms non-compliant chains through hybrid operations, enabling them to pass through the biological constraint screening smoothly. Based on the data in Table 3 and Eq. 7, the encoding time increments are shown in Table 4. Smaller increments indicate more stable encoding times, while larger increments indicate greater variations in encoding time. DNA Fountain exhibits a relatively stable time increment, with an increment ranging from 11 to 12 when transitioning from small to large data volumes. Although YYC maintains relatively low increments between 2 Kbytes and 20 Kbytes and 20 Kbytes to 200 Kbytes, the increment significantly increases to 47.6 when processing data volumes between 200 Kbytes and 2,000 Kbytes, far exceeding DNA Fountain’s maximum increment of 12. On the other hand, the proposed DNA storage method demonstrates a relatively stable encoding time increment, ranging from 4.5 to 8, which is lower than both DNA Fountain and YYC’s time increments. Therefore, when storing larger text files, it is inferred that the proposed DNA storage method is the best choice, with the lowest increment, followed by DNA Fountain and YYC being the least favorable option.

The above analysis shows that the encoding time of this scheme is lower than that of the two storage schemes, DNA Fountain and YYC when storing text with less than 2,000 Kbytes of data. And due to this scheme’s lower and more stable time increase, it is presumed that this scheme is also better than the other two storage schemes when storing text with more than 2,000 Kbytes of data.

### 3.5 Biological experiment

The experimental storage of the Chinese text “Spring” by Zhu Ziqing consists of 747 characters, including punctuation. For the experimental design, each DNA sequence stores 94 characters, resulting in a DNA sequence length of 94 × 7 = 658 bp, corresponding to the data length of 658 bp. The address index (index) is 5 bp, and the length of the mod is designed to be 4 bp, while the iterate is set to 5 bp. The RS code is used for error correction and has a length of 18 bytes, equivalent to 72 bp, allowing for the correction of up to 9 positions of substitution errors, and it cannot correct insertion and deletion errors. The total length of the synthesized DNA sequence is 5 + 658 + 4 + 5 + 72 = 744 bp. After the calculations, the Chinese text “Spring” containing 747 characters is encoded into 8 DNA sequences, with each DNA sequence containing 744 bp. The information capacity of this encoding is measured at 3.01 bits/nt.

**FIGURE 6:**
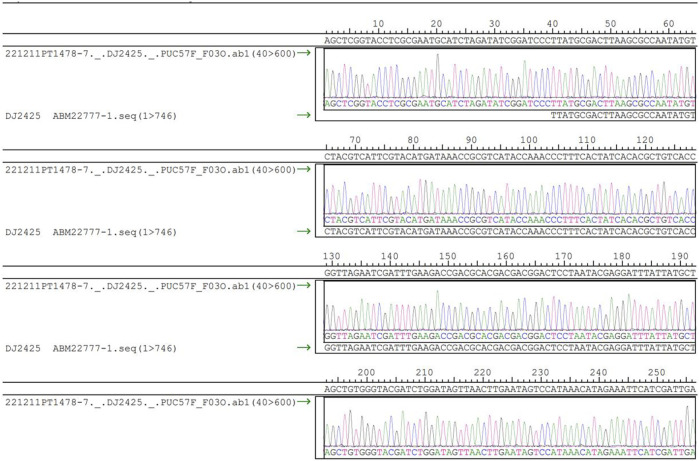
DNA sequencing diagram.

The DNA sequences were synthesized and inserted into plasmids, and before synthesis, the plasmids underwent thorough testing to ensure compliance with the encoding requirements. Taking the first plasmid as an example and the accompanying restriction enzyme electrophoresis gel. Sanger sequencing was employed to verify the consistency between the inserted sequences and the reference sequence. Visually, the cultured plasmids appeared transparent, colorless, and free of impurities or precipitates. The OD:260/280 ratio was measured at 1.859, within the accepted range of 1.8–2.0. Additionally, the OD:260/230 ratio was determined to be 2.375, also meeting the required standard. The identification results of the restriction enzyme project are presented in Figure 6. The gel electrophoresis used 100–10,000 base pair markers for comparison, applying a voltage of 7V for 45 min. Upon comparing the band patterns on the gel with the markers, it was observed that in the first lane, brighter bands appeared between 700 bp and 800 bp, aligning with the designed DNA sequence length of 744 bp, falling within this range. The bands in the second lane were located between 500 bp and 600 bp and 1200 bp and 1500 bp, respectively, corresponding to two types of restriction enzymes. The electrophoresis analysis confirmed that the design structure met the validation criteria. Furthermore, no extra bands were observed in the restriction enzyme electrophoresis gel, indicating the absence of residual RNA and genomic DNA, which also complied with the validation criteria.

After detecting that all the plasmids were compliant, these plasmids were sequenced (Pennisi, 2012), and the sequencing results were all compliant, taking the first plasmid as an example. The sequencing results are shown in Figure 6 below.

The step of synthesizing the DNA sequences was proved to be successful by testing the plasmids, and the synthesized products were verified that the insert sequences were consistent with the reference sequences. By sequencing the DNA sequences and observing the sequencing results and peak maps of these sequences, the use of DNA synthesis technology and one-generation sequencing ensures highly accurate synthesis and sequencing of DNA strands.

## 4 Discussion

### 4.1 Chinese character storage

Chinese is the most complete document character set in the world, covering a long historical time span and containing a large volume of data, making it the most significant research target for storage. Currently, the widely used computer encoding scheme for Chinese Hanzi documents is UTF-8 (8-bit Unicode Transformation Format), with a capacity of 24 bits and accommodating 16,777,216 characters. However, the commonly used Chinese characters comprise only a scale of 5,000 to 7,000 characters. Adopting the UTF-8 encoding scheme results in a tremendous waste of information space. To enhance information capacity, we have selected the commonly used Chinese character library GB2312, which includes 7,445 Chinese characters. By using 7 bases to correspond to one character, we still have an additional 8,939 redundant spaces. For rare or special characters not covered by GB2312, the encoder establishes new corresponding relationships for these characters, expanding the character library to enable their proper storage by comparison with traditional methods (UTF-8), the storage capacity of DNA-GB2312 is shown in Table 5. Additionally, during the Chinese storage process, the grammar rules of Chinese are analyzed, and in case of errors, targeted error correction can be performed in the grammar process.

**TABLE 5:** Comparison of experimental results of DNA storage schemes.

	Information scale (MB)	Information density (bits/nt)	Non-storage space (%)	Error correction	Error correction
Church et al. (2012)	0.650	0.83	17.00	-	NO
Bornholt et al. (2016)	0.150	0.88	44.3	-	NO
Grass et al. (2015)	0.080	1.14	35.96	Reed–Solomon	YES
Blawat et al.	22.000	0.92	42.50	forward error correction	YES
Erlich and Zielinski (2017)	2.110	1.57	20.71	Reed–Solomon	YES
Goldman et al. (2013)	0.630	0.33	79.11	Quadruple redundancy	YES
This work (DNA shift)	0.002	3.01	11.56	Reed–Solomon	YES

### 4.2 DNA shift encoder

In the process of biological storage, to avoid errors during DNA synthesis, sequencing, and PCR processes, the synthesized DNA strands need to adhere to the constraints imposed by biology. In order to satisfy these biological constraints, there are currently two common solutions: code table-based and balanced approaches. The code table-based solution involves designing a code table with specific rules to ensure that the generated DNA bases meet biological constraints, such as Base 64 encoding. On the other hand, the balanced approach involves randomly shuffling DNA sequences and using random sequences for hybridization to meet biological constraints, such as hash functions, linear feedback shift registers in fountain codes, and others.

The code table approach relies on a fixed mapping relationship, enabling efficient synthesis. However, the code table sacrifices some storage space, reducing information encoding density. The balanced methods may require additional storage space for displacement registers, pseudo-random number generator seeds, or corresponding identifiers. Moreover, since the balanced methods are generally based on randomness, the generated results may not always pass the biological constraints, necessitating the need for result filtering. Non-compliant sequences are usually discarded, and new sequences are generated in their place. This can lead to a concentration of computational efforts on generation and filtering, resulting in lower coding efficiency.

The DNA shift encoder used in this paper is to realize the biological constraints utilizing equalization. Due to the simple principle of the adopted encoder, the strategy of transforming bases by position can effectively avoid the generation of homopolymer. At the same time, the DNA shift algorithm possesses high efficiency due to its low complexity.

### 4.3 Error correction capability analysis

In order to validate the feasibility of the DNA coding storage scheme proposed in this paper through biological experiments, the experimental results of this study are compared with other classical DNA storage schemes that have been verified through biological experiments. The performance parameters are shown in Table 3. The “Storage Data Volume” refers to the amount of data successfully stored in biological experiments. It should be noted that the “Information Capacity” in this table differs from the theoretical information capacity used in Chapter 3; it represents the actual information capacity, considering factors such as address encoding and error correction. This study also considers the encoding factors of “mod” and “iterate” where the actual information capacity is calculated as the total number of bits in the stored files divided by the total number of bases. The “Invalid Ratio” represents the ratio of bases in the synthesized DNA sequences that do not contain stored data to the total number of bases. The “Recovery” indicates whether the decoded file obtained from the DNA sequencing of bases matches the original file. From the table, it can be observed that the DNA storage scheme proposed in this paper exhibits higher information capacity and a lower invalid ratio when storing Chinese text compared to other schemes. However, it should be noted that the use of the RS error correction scheme can only correct substitution errors and cannot address insertion and deletion errors. Therefore, the DNA storage scheme proposed in this paper outperforms other storage schemes regarding both the invalid ratio and information capacity when storing Chinese text.

### 4.4 Plasmid biorepository

The DNA single-stranded storage’s challenges include various errors, such as strand breaks, rearrangements, and indels that frequently arise during DNA synthesis, amplification, sequencing. Song et al. (2022) proposed a *de novo* strand assembly-based strategy (DBGPS) that exhibits exceptional information recovery capabilities. Specifically, it successfully retrieved 6.8 MB of data from a severely corrupted sample. Moreover, DBGPS demonstrates a logical density of 1.30 bits/cycle and a physical density of 295 PB/g. The plasmid exists in the form of a long double-stranded structure. It is precisely because of this structure that the plasmid scaffold has self-checking capabilities, which are of significant importance for its stability during storage and the accuracy of bidirectional sequencing. Compared to addressing strategies based on short-chain DNA, the plasmid allows for an extended effective information encoding payload. Additionally, reducing the number of non-information encoding regions, such as addressing blocks, increases the information storage density correspondingly. Under the protection of the plasmid, our DNA chain can grow as long as possible while its stability and sequencing accuracy are further improved. Each nucleotide can be fully utilized for information storage. Moreover, using the plasmid-based scaffold, along with the addition of a protein shell, provides better and more stable storage. It also exhibits a natural affinity with living organisms, allowing the use of *Escherichia coli* as a preparation factory to achieve low-cost and efficient completion of information storage and replication tasks. For *in vivo* DNA storage, there are two challenges. Firstly, the DNA molecules may undergo cellular replications leading to their enrichment. Secondly, the presence of artificial sequences can potentially interfere with the natural sequences. In addressing this issue, Song and Zeng (2018) proposed a self-error-detecting, three-base block encoding scheme (SED3B), which exhibits high error tolerance and low biological relevance, rendering SED3B highly promising for the orthogonal encoding of information in living cells. Furthermore, the plasmid offers advantages in terms of sequencing accuracy, data stability, and security. Hence, plasmid-based storage facilitates achieving high precision and high storage density for the DNA shift encoding algorithm.

## Data Availability

The original contributions presented in the study are included in the article, further inquiries can be directed to the corresponding author.
